# Viability, Sublethal Injury, and Release of Cellular Components From *Alicyclobacillus acidoterrestris* Spores and Cells After the Application of Physical Treatments, Natural Extracts, or Their Components

**DOI:** 10.3389/fnut.2021.700500

**Published:** 2021-08-11

**Authors:** Antonio Bevilacqua, Leonardo Petruzzi, Barbara Speranza, Daniela Campaniello, Emanuela Ciuffreda, Clelia Altieri, Milena Sinigaglia, Maria Rosaria Corbo

**Affiliations:** Department of Agriculture, Food, Natural Resources and Engineering, University of Foggia, Foggia, Italy

**Keywords:** *Alicyclobacilli*, proteins, calcium dipicolinate, inactivation, not-lethal effect

## Abstract

*Alicyclobacillus acidoterrestris* is a spoiling microorganism regarded as one of the most important causes of spoilage of fruit juices and acidic products. In this paper, four strains of *A. acidoterrestris* (type strain-DSM 3922; two wild strains isolated from soil-C8 and C24; wild strain isolated from a spoiled pear juice CB1) were treated through natural extracts/active compounds from essential oils (EOs), and physical treatments were used to assess their susceptibility and the presence of sublethal injury. The characterization of damage was also performed. The results suggest that it is possible to control *A. acidoterrestris* through alternative approaches, although the effect relied upon the age of spores. In addition to the mere antimicrobial effect, some treatments could cause a sublethal injury on spores. Lemon extract was the most effective treatment for both the antimicrobial effect and the sublethal injury, as evidenced by the release of proteins, and calcium dipicolinate [dipicolinic acid (DPA)] by fresh spores and only DPA (with an exception for C8) by old spores. A sublethal injury with protein release was also found for physical treatments [US (ultrasound) or heating]. For the first time, this paper reports on the existence of a sublethal injury for *A. acidoterrestris*, and this evidence could also be a challenge, because injured microorganisms could restore their metabolism, or an opportunity to design new preserving treatments.

## Introduction

*Alicyclobacillus acidoterrestris* is a spoiling microorganism regarded as one of the most important causes of spoilage of fruit juices and acidic products; thus, it is a control target for thermal treatment effectiveness (1). It represents a threat of spoilage in acidic foods because spores can germinate and grow at low pH (2). *A. acidoterrestris* does not produce gas during growth, and only little changes occur in juices (turbidity and/or white sediment at the bottom of the container). The microorganism produces a taint compound, identified as guaiacol, responsible for an offensive smelling described as smoky, antiseptic, or disinfectant-like flavor (3). Nevertheless, guaiacol off-taste is a defect that the beverage industry tries to avoid, and consumers could easily detect in the final product (4).

Pasteurization of fruit juices is generally used to control bacterial contamination; the general rule by U.S. Food and Drug Administration is 5-log pathogen reduction in the juice through thermal or equivalent treatments (5). Fruit juices generally undergo a flash treatment (temperature/short time) to avoid loss in organoleptic and nutritional properties (6, 7), but *A. acidoterrestris* spores can survive (8, 9).

Some technologies that could be used to control *A. acidoterrestris* are high-pressure homogenization (HPH), ultrasound (US), or essential oils (EOs), extracts, and their active compounds. HPH was proposed as a tool for the inactivation of alicyclobacilli some years ago (10). Generally, HPH could affect the cell viability by the disruption of cell wall (11), inducing a structural rearrangement of proteins, which in turn causes the exposure of their hydrophobic regions and the disaggregation of their supramolecular structure (11). On spores, homogenization pressure causes the release of dipicolinic acid (DPA) and ions, which are responsible for core hydration, spore germination, and subsequently their inactivation (12, 13).

Ultrasound is another non-thermal process; it could disrupt bacterial clusters and kill cells through acoustic cavitation, localized heating, and free radical production (14, 15); however, Ojha et al. (16) postulated a unifying mechanism called sonoporation to elucidate the mode of action of sonic waves on cells.

Essential oils, extracts, and EO components are active against alicyclobacilli and could induce variable levels of spore or cell reduction (17–19). Cinnamaldehyde (100–500 ppm) inhibited *A. acidoterrestris* for at least 13 days (20), and eugenol could reinforce its action (21). Citrus extract was proposed as a suitable natural preservative for many microorganisms (22–24), but few data are available on the spores of *A. acidoterrestris*.

Chemical or physical treatments could have a different effect on microorganisms (inactivation, sublethal injury, or no effect) (25). Sublethal damage in spores could be a significant threat due to the complexity of the spore entity and its intrinsic high resistance to stress. Sublethally damaged microorganisms could repair injury and return to a normal physiological state; some data on foodborne microorganisms suggest that they can return a normal physiological state after 12 h (26). However, few results are available on sublethal injuries on spores, namely, on *A. acidoterrestris*.

The goal of this paper was to evaluate the effect of natural extracts (lemon extract and citrus extract) or component from EOs (eugenol and cinnamaldehyde), as well as heating, HPH, and US on four strains of *A. acidoterrestris* (DSM 3922T, CB1, C8, and C24), by evaluating different trends among fresh and old spores, cells, and activated spores regarding the extent of inactivation, the occurrence of sublethal injury, and the signs of injury.

## Materials and Methods

### Strains and Culture Conditions

The following strains were used: (i) *A. acidoterrestris* DSM 3922^T^ from Deutsche Sammlung von Mikroorganismem und Zellkulturen's collection, Braunschweig, Germany; and (ii) *A. acidoterrestris* C8, C24, and CB1, belonging to the collection of the Department of Agriculture, Food, Natural Resources and Engineering (Foggia University), and isolated from soil and a spoiled pear juice (27). The strains of *A. acidoterrestris* were stored at 4°C on malt extract agar (MEA) (Oxoid, Milan, Italy) and acidified to pH 4.5 through a sterile solution of citric acid (1:1, w/w). Working cultures were prepared by inoculation with 5–6 log cfu/ml in 5 ml of fresh acidified malt extract broth (MEb) and incubated at 45 ± 1°C for 24 h.

### Spore Suspension

A spore suspension of each strain was produced on acidified MEA, through cultivation at 45 ± 1°C for 7 days, surface washing, and centrifugation for three times of spore suspension at 1.000 x g for 10 min, as reported by Bevilacqua et al. (20). Spore suspension (30 ml in plastic tube of 50-ml Falcon plastic) was heated at 80°C for 10 min to eliminate vegetative cells, put in ice, and stored at 4°C. Spore number was assessed through the spread plate count on acidified MEA, incubated at 45 ± 1°C for 2 days, and reported as log cfu/ml.

### Treatments

The experiments were performed on fresh spores (produced and used within 2 weeks), old spores (spores stored at 4°C for 4 months before their use), activated spores (activation of spores was done at 70°C for 10 min), and cells (microorganism grown for 24 h at 45°C).

#### Extracts and Compounds

Eugenol (MP Biomedicals, Aurora, Ohio), cinnamaldehyde (ICN Biomedicals, Aurora, OH), lemon extract (the extract was produced from the peel of *Citrus limonum*; the exact composition is not known as the extract is covered by a patent; however, the amount of limonene was >10% and other components were bioflavonoids, ascorbates, and polyphenols) (Spencer Food Industrial, Amsterdam, The Netherlands), and citrus extract [produced from grapefruit, sweet orange, and tangerine; the composition was as follows: ascorbic acid and ascorbates, linked with citrus bioflavonoids, 4.0–7.20%; hydrated glycerin linked with other traces of citrus polyphenols, carbohydrates, bio-flavoproteins, pectin, citrus sugars, citric acid, 30.80–36.60%; water, 6.00–11.00%. The amount of limonene was <5% (Biocitro®, Quinabra, Probena, Spain] were used in this study. Pure compounds/extracts were stored in dark at room temperature (eugenol and cinnamaldehyde) or at 4°C (extracts). Stock solutions (25,000–50,000 ppm) were freshly prepared before each use in ethanol–water (1:1, v/v) for eugenol, cinnamaldehyde, and lemon extract, or in distilled water for citrus extract, and sterilized by filtering through membranes (0.2 μm, Millipore, Milan, Italy).

#### Physical Treatments

High-pressure homogenization treatment was done at 150 MPa for one, two, or three times; a pilot equipment was used (PANDA 2K, Niro Soavi s.p.a., Parma, Italy). Before each experiment, the equipment was washed through sterile distilled water (70°C) and sterile water at room temperature (20°C). After the treatment, the samples were collected into 100-ml sterile tubes and immediately cooled at 4°C in ice.

For US, the treatment was done on 30 ml samples put in 50-ml plastic tube for 6 or 8 min (pulse set to 4 s) through a VC Vibra Cell US equipment, model VC 130 working at 130 W/20 kHz (Sonics and Materials Inc., Newtown, CT, United States); the power was set to 40% or 60% of the maximum intensity. Before each treatment, the ultrasonic probe was washed with sterile distilled water, and immediately after processing, the sample was cooled in ice. The probe (5 × 60 mm; diameter × the active component of horn) was put 2–3 cm below the surface of water.

Heat treatment (30 ml samples in 50-ml plastic tube) was performed at 95°C in a water bath under static conditions; immediately after the treatment, the samples were cooled in ice.

#### Experiments

All targets of *A. acidoterrestris* were subjected to chemical or physical treatments.

- Chemical treatment consisted of saline solution (0.9% NaCl) inoculated to 5–6 log cfu/ml and supplemented with eugenol (500 ppm), cinnamaldehyde (250 ppm), lemon extract (250 ppm), or citrus extract (250 ppm).
- Physical treatment consisted of saline solution, inoculated to 5–6 log cfu/ml, treated at 150 MPa for one, two, or three times (HPH), 60% of power for 6 min/pulse 4 s (US), or at 95°C for 5 min (heating).

For each treatment, a control was used (saline solution inoculated with alicyclobacilli but not treated through chemical or physical processing). The viable count was assessed both on the optimal laboratory medium (non-selective medium) and on the restrictive one (selective medium with salt), after treatment application (T0), 1 day (T1), and 2 days (T2) at 45 ± 1°C.

The results from these experiments were used to evaluate the “antimicrobial effect,” that is, the decrease in viable count compared to the control for each time of sampling.

After the application of different stress, damage detection was performed and reported as “percentage of sublethal injury,” evaluated as follows (28):

(1) $$\begin{array}{r} \%Sublethal\;Injury\;=\;\frac{counts\;on\;NSM-counts\;on\;SM}{counts\;on\;NSM}\times 100 \end{array}$$

The counts on the formula were used as log CFU/ml values.

NSM: non-selective medium; acidified MEA (pH 4.5), incubated at 45°C for 48 h.

SM: selective media; acidified MEA+0.5% NaCl (DSM 3922), +1.0%NaCl (CB1 and C8), +0.8% NaCl (C24). The scheme to point out a sublethal injury is in the S1 in Supplementary Material.

### Injury Characterization

Sublethal injury on fresh and old spores of *A. acidoterrestris* was studied after the application of selected treatments: lemon extract (250 ppm), US (40% 6 min 4 s), heating (95°C for 5 min), immediately after the treatment (T0) or after 2 days (T2) of incubation at optimal culture conditions, through bovine serum albumin (BSA) protein assay and colorimetric assay for DPA in bacterial spores.

#### Bovine Serum Albumin Protein Assay

Untreated and treated samples (5–6 log cfu/ml) were centrifuged at 6,000 x g for 10 min. Then, 2.0 ml of BSA working reagent (BSA Protein Assay Reagent Kit, Sigma-Aldrich) was added to 0.1 ml of supernatant and incubated at 60°C for 15 min. After that, the absorbance at 562 nm was determined [spectrophotometer UV-VIS DU 640 Beckman (Fullerton, CA)]. The calibration curve was built by using BSA as a standard (29).

#### Colorimetric Assay for DPA in Bacterial Spores

After chemical or physical treatments, spores were heated at 121°C, cooled, acidified with 0.1 ml of 1.0 N acetic acid, and left at room temperature for 1 h. After a centrifugation at 1,500 g x 10 min, 4 ml of supernatant was mixed with 1 ml of 1% Fe (NH4)2(SO4)2?6H20 (Sigma-Aldrich) and 1% of ascorbic acid in 0.5 M sodium acetate buffer solution. DPA was measured as absorbance at 440 nm. Pure DPA (Sigma-Aldrich) was used as the calibration standard (12).

### Statistics

All experiments were performed at least on two independent batches; each analysis was repeated. The significant differences between selective and nonselective media were determined using Student's *t*-test (*p* < 0.05) using the software Statistica for Windows (Tulsa, Oklahoma). Moreover, all data were submitted to one-way ANOVA and factorial ANOVA and to Tukey's test (*p* < 0.05). The kind of strain, the treatment, and the sampling time were used as independent factors (predictors).

## Results and Discussion

### Antimicrobial Effect

The use of EOs, natural extracts, and their active components was suggested as a mean to delay or inhibit *A. acidoterrestris* in acidic foods (30–32), because some natural molecules are extracted from plants and fruits; thus, they are not perceived by consumers as chemicals, but as a part of product or natural compounds.

**Figure 1:**
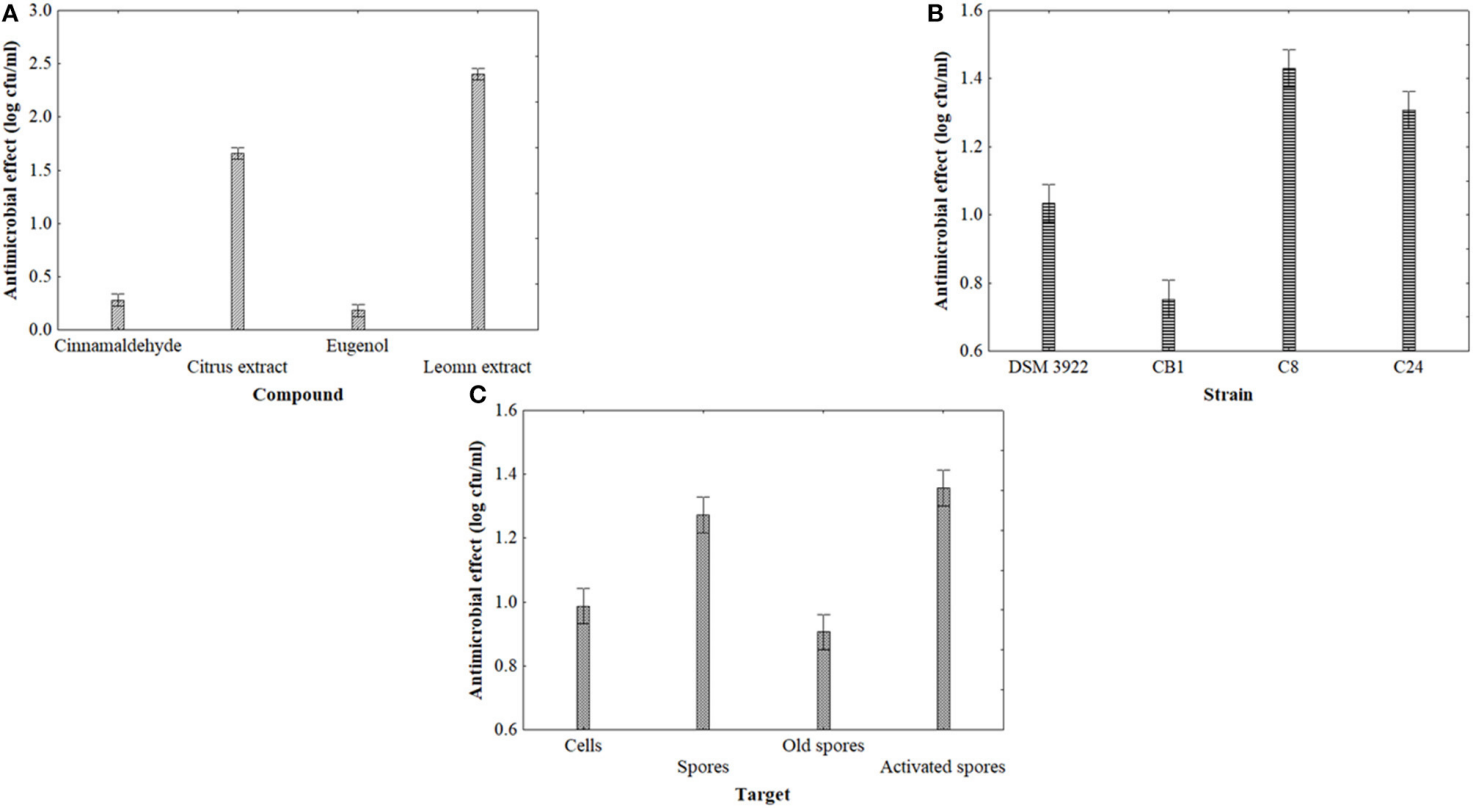
Decomposition of the statistical hypothesis of ANOVA for the effect of compounds **(A)**, strain **(B)**, and target **(C)** on the antimicrobial effect (reduction of viable count, log cfu/ml) of *A. acidoterrestris*. Vertical bars denote the 95% confidence intervals.

Figure 1 shows the statistical effects of the kind of the antimicrobial compound (1A), strain (1B) and targets (1C). The antimicrobial effect was strain dependent and relied on the kind of target (new, old, activated spores or cells); moreover, the contact time played a significant role, too (data not shown). Citrus and lemon extracts exerted a significant inhibition (mean reduction of the target of 2 log cfu/ml), while the effect of cinnamaldehyde on alicyclobacilli was slight (Figure 1A). These results were not in line with preliminary results (20). The antimicrobial activity of extracts and natural compounds is probably the result of the bioactivity of phenolic rings, and also the type of alkyl group plays a significant role (33).

Lemon extract is effective toward a wide range of microorganisms (22, 23). The results confirmed these findings; lemon extract exerted a significant effect on all the targets as a single term as well as in interaction with the contact time (data not shown). The last antimicrobial activity studied was citrus extract, as in the past we found some promising results in terms of juice stabilization (34). It is a combination of extracts from grapefruit, sweet orange, and tangerine, but it has some benefits, because it is colorless and odorless and water soluble. Citrus extract was always significant on all the targets, and many times, it played an interactive role with contact time (data not shown).

The antimicrobial activity was related to the strain (Figure 1B) with type strains (DSM 3922) and CB1 more resistant than others. The antimicrobial activity of pure compounds, active molecules from EOs or extracts, is probably the result of the inhibition of the cascade process of spore-to-cell transition (germination, outgrowth, and cell multiplication) in several points; for example, *Bacillus subtilis*, after the exposure to clove oil, could germinate but it is not able to start outgrowth (35). Finally, a significant inhibition was recovered for spores and activated spores (Figure 1C). As expected, old spores were more resistant, which was in agreement with Sokolowska et al. (36).

For the results for extracts, mainly for lemon extract, it is well-known that the antimicrobial activity of an EO or an extract is the result of both major (components at high concentrations) and minor (low amounts) compounds (33). Minor compounds could exert a strengthening effect and improve the performances of major compounds (30, 33). This hypothesis is strengthened by the fact that limonene, the major component of lemon extract, in the past did not show a significant effect on the outgrowth of *A. acidoterrestris* (20). However, it is also important to point out the different conditions and strains (at least some) used for the experiments: In the current paper, the assays were done in a saline and isotonic solution (0.9% NaCl), where the integrity of spores and cells is preserved by osmotic shock. In this medium, outgrowth (passage from spores to cells) and growth (for vegetative cells) are not possible, while in other papers where the same strains and extract were studied, the experiments were done in laboratory media containing nutrients (34) and the presence of nutrients could modify the bioactivity of extracts and the ability of spores and cells to repair injury and start the growth or outgrowth. The choice of the kind medium for such assays is a critical step; the idea beyond the use of saline solution is to avoid the masking effect of complex media and to point out also low levels of inactivation and sublethal injury.

For the other pure compounds (eugenol and cinnamaldehyde), the amounts used in this research, as also stated elsewhere (20), could not have a practical application in foods because such high amounts cause a strong organoleptic impact; however, the aim of this paper was to focus on the existence of a sublethal injury on cells and spores.

Thermal processing is the traditional method to delay or inhibit *A. acidoterrestris* in acidic drinks (37), but some non-thermal methodologies for food preservation could be of interest, because they could achieve a dual effect (inactivation of spoiling or pathogenic microorganisms and retention of sensorial and nutritional quality) (38–40).

**Figure 2:**
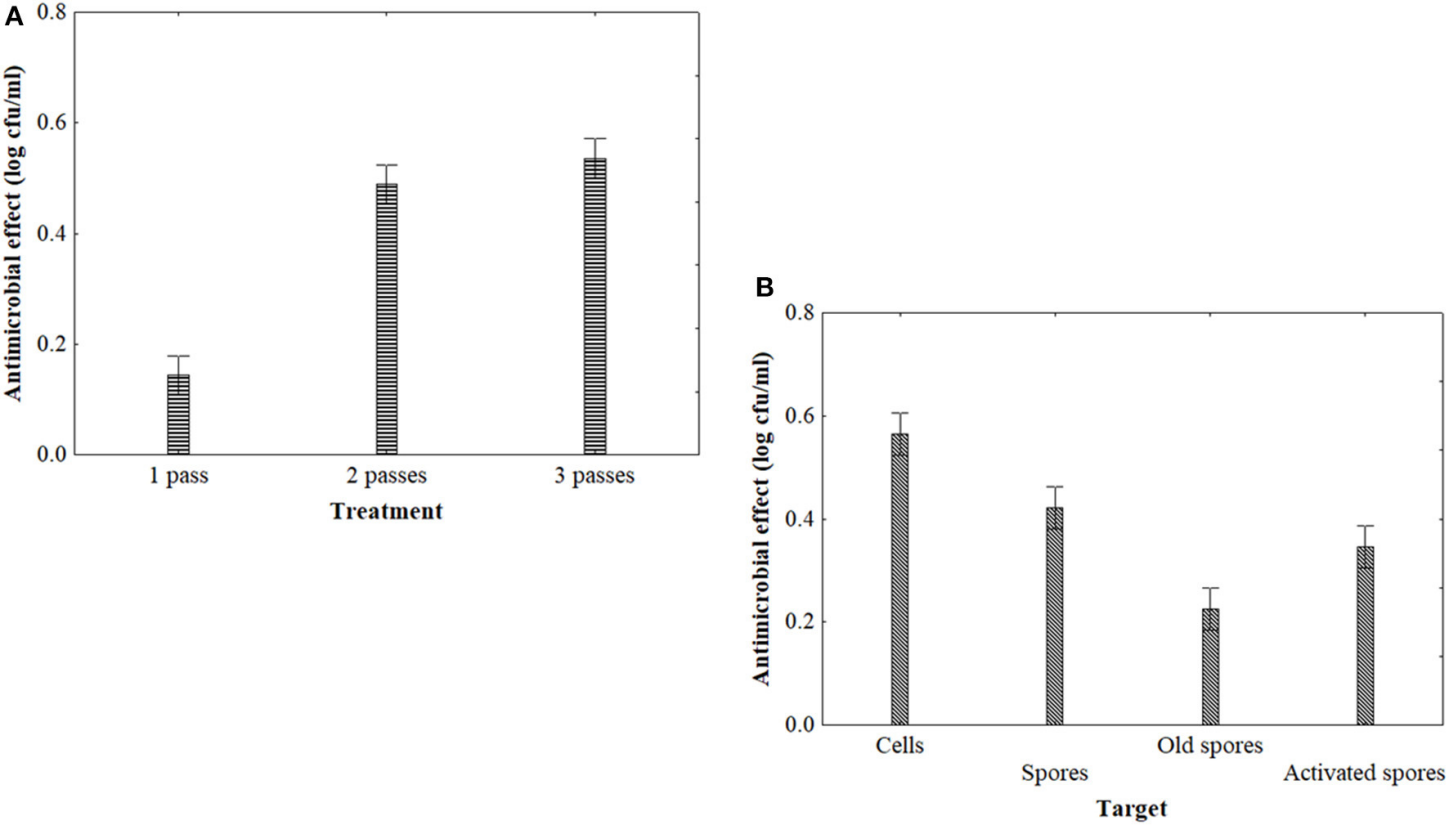
Decomposition of the statistical hypothesis of ANOVA for the effect of homogenization on the antimicrobial effect (reduction of viable count, log cfu/ml) of *A. acidoterrestris*. Vertical bars denote the 95% confidence intervals. **(A)**, effect of the treatment (one pass, single treatment at 150 MPa; two and three passes: 2 and 3 treatments at 150 MPa); **(B)**, effect of target.

Three kinds of treatments were analyzed in this study: HPH, US, and heating (used as reference). Concerning homogenization, the statistical analysis pinpointed a significant effect of passes, target (old or fresh spores, cells), and time (data not shown). The HPH effectiveness relies on several process parameters or microbial–physiological factors, as well as on the characteristic of the treated product (41). Although the statistical analysis revealed a significant effect of two and three passes (Figure 2A), spores appeared very resistant, as previously reported by Bevilacqua et al. (42).

As expected, the cells were more sensitive than spores (old spores) (Figure 2B), probably due to a lower exposure of proteins in spores and due to the presence of dipicolinic acid. Moreover, the resistance of the spores is not surprising, as they are very resistant to heat and high hydrostatic pressure treatments (43).

Alicyclobacilli were also processed through US, by using different combinations of power and time. US acts through sonoporation on microorganisms (16), with multiple effects, including cavitation, production of free radicals, mechanical injuries, and acoustic streaming (14, 16, 38, 44). Cavitation causes both a local increase in temperature and a mechanical injury (45). Moreover, water molecules are the sources of H- and OH- free radicals, which in turn cause the biological effect on DNA and proteins (46).

**Figure 3:**
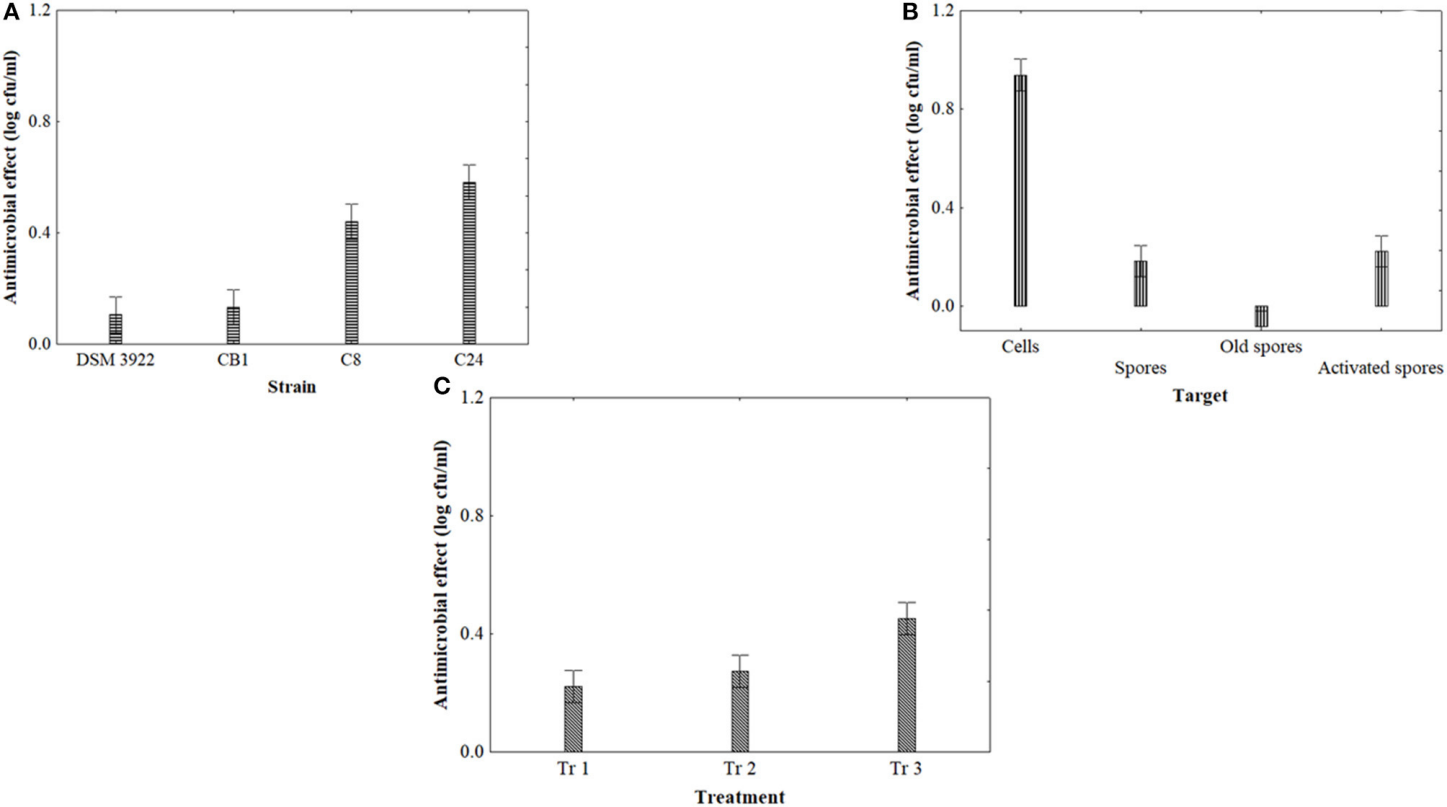
Decomposition of the statistical hypothesis of ANOVA for the effect of US on the antimicrobial effect (reduction of viable count, log cfu/ml) of *A. acidoterrestris*. Vertical bars denote the 95% confidence intervals. **(A)**, effect of strain; **(B)**, effect of target; **(C)**, effect of the treatment (tr 1, 40% 6 min 4 s; tr 2, 60% 6 min 4 s; tr 3, 40% 8 min 4 s).

Figure 3 shows the output of statistical analysis for US. The kind of strain (3A) and the kind of target (3B) were found to be significant, along with the effect of the treatment (3C). Generally, the strains C8 and C24 were more affected by US with a mean reduction of targets by 0.6–0.8 log cfu/ml (Figure 3A). As expected, spores were more resistant, while cells were significantly affected (mean reduction of 1 log cfu/ml). However, spore activation did not play a significant role, as activated spores experienced the same trend than fresh and old spores (Figure 3B). Finally, the strongest treatment was the treatment 3, characterized by the highest duration (8 min) (Figure 3C).

Thermal treatments were also used in this work, since they are still the most common way to control alicyclobacilli in juice (34). Depending on the strain and isolation source, thermal resistance of spores is greatly variable; for example, there are some strains that possess a strong intrinsic resistance to temperature: Goto et al. (47) reported for *A. acidoterrestris* strain AB-1 a D${}_{89^{∙}C}$ of 10.9–13.7 min, whereas D${}_{95^{∙}C}$was determined to be 2.1–3.2 min.

The antimicrobial effect of the thermal treatment was very low (0.2–0.4 log cfu/ml), thus confirming thermal resistance of *A. acidoterrestris*. Moreover, the differences among the targets (at least for spores) were not significant (data not shown).

### Sublethal Injury and Injury Characterization

The occurrence of sublethal injury was studied only on some combinations, that is, the addition of lemon extract (250 ppm), or the application of US (40% for 6 min 4 s) or heating (95°C for 5 min). HPH was not tested in this step because of the low antimicrobial effect, while for US the treatment for 6 min was used because the decomposition of the statistical hypothesis for the antimicrobial effect showed a similar trend for all strains, while the treatment for 8 min determined strong differences among the strains, which could affect the recovery of a sublethal injury (data not shown).

There are several definitions of stress, but generally there is an input, a black box, and an output. The input is environment, a treatment, or other factors; the box is the microorganism itself, a dynamic environment which as a response to a stress (input) is subjected to a change in genome, or proteome. The output could be different (reduction of growth rate, inactivation, sublethal injury), because microorganisms are dynamic entities (48, 49).

After an injury, cells react through morphological, physiological, or biochemical processes (50). There are several targets for injuries; the most important ones are cell wall, cytoplasmic membrane or inner membrane, ribosomes, DNA, RNA, tricarboxylic acid cycle enzymes as well as many other enzymes (51). In addition, injured microorganisms are more sensitive to antimicrobial agents contained in selective media; thus, their growth could be delayed or inhibited on such substrates, but these microorganisms retain their pathogenicity and spoiling impact (52). Under favorable conditions, injured microorganisms can resuscitate and repair damage (52). Thus, the detection of sublethally injured bacteria is critical to quality control procedures performed in the food processing industry (53).

An excellent method available for detecting and enumerating sublethally injured bacteria is the application of selective culturing procedures, since sublethally injured bacteria become sensitive to salt or other selective compounds due to damage in their membrane and modifications of their permeability and lose their ability to grow on selective media (26, 52). The amounts of salt for the medium to detect sublethal injury were chosen after a preliminary experiment aimed at assessing the NIC (not inhibitory concentration) of salt, that is, the highest concentration of salt not affecting the growth of not-injured *A. acidoterrestris*. Some details are in the S1 in Supplementary Material.

Table 1 shows the samples where a sublethal injury was detected. Injury was mainly detected after the application of extracts and active components; the old spores of C8 experienced an injury immediately after the addition of eugenol (8.54%), while the injury was detected on the spores of C24 after 1 or 2 days (7.18% on fresh spores and 28.89% on activated spores). Cinnamaldehyde caused a sublethal injury in the type strain DSM 3922 (cells and activated spores, 10.14% and 4.76%), CB1 (cells, 7.62%), and C24 (old spores, 3.80%). Lemon extract caused the highest extent of sublethal injury and the fresh spores were always affected (from 13.75% in C8 to 31.71% in DSM 3922); other targets were also affected, for example, old spores in C8 (51.15%) and activated spores in CB1 and C8 (14–17%). An evidence of a possible sublethal injury was also found for citrus extract.

**Table 1:** Sublethal injury (%) on *A. acidoterrestris* caused by eugenol (500 ppm), cinnamaldehyde (250 ppm), lemon extract (250 ppm), and citrus extract (250 ppm) immediately after application (T0), or after 1 (T1) or 2 days (T2) of incubation at 45 ± 1°C.

	**DSM 3922**	**CB-1**	**C8**	**C24**
**Eugenol**				
Cells	/^*^	/	/	/
Fresh spores	/	/	/	7.18 ± 1.58 (T1)
Old spores	/	/	8.54 ± 1.41 (T0)	/
Activated spores	/	/	/	28.89 ± 5.56 (T2)
**Cinnamaldehyde**				
Cells	10.14 ± 1.41 (T2)	7.62 ± 0.97 (T0)	/	/
Fresh spores	/	/	/	/
Old spores	/	/	/	3.80 ± 0.01 (T2)
Activated spores	4.76 ± 0.14 (T2)	/	/	/
**Lemon extract**				
Cells	/	/	/	/
Fresh spores	31.71 ± 0.001 (T0)	17.88 ± 1.69 (T0)	13.75 ± 1.02 (T1)	16.71 ± 3.76 (T0)
Old spores	/	/	51.15 ± 9.04 (T2)	/
Activated spores	/	13.84 ± 3.70 (T2)	17.01 ± 2.88 (T2)	/
**Citrus extract**				
Cells	/	10.87 ± 0.74 (T2)	/	/
Fresh spores	/	/	/	/
Old spores	/	/	/	9.20 ± 0.86 (T2)
Activated spores	/	8.71 ± 1.87 (T2)	/	/

*Mean values ± standard deviation*.

* *Sublethal injury not found*.

Finally, spores did not experience or experienced a slight injury after some physical treatments (old spores of CB1 after three pass-treatments, 18.56%) (data not shown). After the qualitative evaluation of a sublethal damage, injury was characterized at different levels, that is, the release of DPA or proteins.

Injured cells lose some as amino acids, proteins, and nucleic acids through leakage into their surroundings (54, 55), since the cell membrane is generally disturbed by stresses (56). On the other hand, the release of DPA is frequently associated with germination of spores after the activation of the nutrient receptors. It is generally accompanied by the activation of lytic enzymes of spore cortex, which are responsible for the degradation of the cortex (57). However, the release of DPA could be a sign on an injury occurring on spores (49, 58), as also suggested by Chaves-Lòpez et al. (12) after pressurization conditions.

Table 2 shows the release of proteins and DPA from fresh and old spores. In fresh spores, the addition of lemon extract determined the release of proteins (DSM 3922 and C8) or DPA (CB1 and C24); spores were also affected by US (C8, release of proteins and DNA).

**Table 2:** Release of proteins and DPA from fresh and old spores of *A. acidoterrestris* after lemon extract (250 ppm) addition (lemon), US treatment (40%-6 min-4 s) (US), or heating at 95°C for 5 min (heat).

**Strain**	**Treatment**	**Release of**
**Fresh spores**		
DSM 3922	Lemon-T0	Proteins (2.70 ± 0.84 ppm)
CB-1	Lemon-T0	DPA (2.65 ± 0.66 ppm)
C8	Lemon-T0	Proteins (1.02 ± 0.34 ppm) and DPA (12.65 ± 0.88 ppm)
	US-T0	Proteins (14.35 ± 0.63 ppm), and DPA (7.50 ± 0.66 ppm)
C24	Lemon-T0	DPA (3.12 ± 0.44 ppm)
**Old spores**		
DSM 3922	Heat-T0	Proteins (4.05 ± 0.42 ppm)
C8	Lemon-T2	DPA (18.59 ± 1.99 ppm)
C24	US-T0	Proteins (4.95 ± 0.21 ppm)

*Mean values ± standard deviation*.

*T0, immediately after the treatment; T2, after 2 days*.

The results on old spores showed a general trend: Spores generally did not release DPA after physical treatments (US or heating), and the release of DPA was found only after the addition of lemon extract.

The same treatments used in this research were assessed by authors (58) toward some bacilli (namely, *Bacillus coagulans* and *Bacillus clausii*); the characterization of injury on *B. coagulans* showed that the spores of this microorganism release proteins after the addition of antimicrobial oils and DPA after US and thermal treatments with a few differences between old and fresh spores, thus suggesting some common traits for lemon extracts (release of proteins), as well as differences between bacilli and alicyclobacilli, due to the unique characteristics of the genus *Alicyclobacillus*.

## Conclusions

*A. acidoterrestris* is a threat in fruit juices and acidic products, but the results of this paper confirm that it is possible to control *A. acidoterrestris* through alternative approaches, although a variable to be considered is the age of spores.

Both extracts/active components and physical treatments could cause a sublethal injury on the surviving cells and spores, but it was not possible to point out a general trend because the results were strongly strain dependent. Finally, lemon extract caused the release of proteins, and DPA by fresh spores, with some differences due to the strain; old spores did not release DPA (with an exception for C8), and the release of proteins was a consequence of a physical treatment (US or heating).

For the first time, this paper shows that the spores of *A. acidoterrestris* could experience an injury when treated by chemical or physical treatments and release proteins and DPA; moreover, fresh and old spores behave in a different way, and the trend is affected by the strain. In addition, the results showed similarities and differences with some bacilli tested in the past, thus confirming the unique traits of *Alicyclobacillus* spp.

Further investigations are required to assess the effect of other treatments and if a sublethal injury could occur in real systems, because of the impact of a sublethal injury of microbial stabilization and for the possibility of combining different treatments to have a shift from sublethal injury to an effective inactivation.

## Data Availability Statement

The raw data supporting the conclusions of this article will be made available by the authors, without undue reservation.

## Author Contributions

AB, MS, and MC designed and conducted the research and contributed to the data analysis. AB, LP, BS, DC, EC, and CA collected the data. AB, LP, EC, and MC contributed to the interpretation of results and the manuscript preparation. All of the authors contributed to the review, editing, and approval of the final manuscript.

## Conflict of Interest

The authors declare that the research was conducted in the absence of any commercial or financial relationships that could be construed as a potential conflict of interest.

## Publisher's Note

All claims expressed in this article are solely those of the authors and do not necessarily represent those of their affiliated organizations, or those of the publisher, the editors and the reviewers. Any product that may be evaluated in this article, or claim that may be made by its manufacturer, is not guaranteed or endorsed by the publisher.
