# Supplementary material for: Viability, Sublethal Injury, and Release of Cellular Components From Alicyclobacillus acidoterrestris Spores and Cells After the Application of Physical Treatments, Natural Extracts, or Their Components
Source: Front Nutr. 2021 Aug 11;8:700500. doi: 10.3389/fnut.2021.700500 (PMC8385314; doi:10.3389/fnut.2021.700500)
Supplement: Supplementary file 1 [file Data_Sheet_1.docx]

**S1: Scheme to point out sublethal injury**


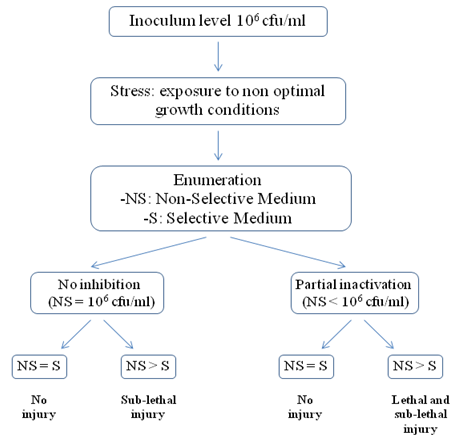


**S2: Determination of selective medium**

**Methodology**

Aliquots of 5 ml of saline solution (0.9% NaCl) were inoculated with 5-6 log cfu/ml of each strain. The selective medium for each microorganism was determined after plate counting on respective optimal culture medium (non-selective medium, Malt Extract Agar acidified to pH 4.5) and on the same medium supplemented with different amounts of NaCl (0.3%; 0.5%; 0.8%; 1%; 2%; 3%; 4% and 5%); both media were incubated at 45 °C for 48 h, 5, and 6 days. The experiments were performed twice, and the results were analyzed through t-test (p<0.05), to point out significant differences between optimal and not optimal medium.

**Results**

The highest concentration of salt not affecting growth and colony dimension was chosen as the amount to add to design restrictive medium to detect sub-lethal inhury.

As an example, table 1 shows the results for a strain.

**Table 1:** Determination of the critical concentration of NaCl for the restrictive medium for the strain CB1 (S, significant difference; NS, not significant).

Data are the average of two repetitions.

| CB1 | 48 h | 5 days | 6 days | Difference with control |
| --- | --- | --- | --- | --- |
| Control | 6.51 | 6.43 | 6.67 | - |
| 1% NaCl | 6.46 | 6.85 | 6.79 | Ns |
| 2% NaCl | 5.91 | 5.52 | 5.52 | S |
| 3% NaCl | 5.71 | 5.51 | 5.53 | S |
| 4% NaCl | no growth | 4.20 | 4.26 | S |
| 5% NaCl | no growth | 3.94 | 3.08 | S |

**Table 2:** Amounts of salt for the restrictive media.

|  | non-selective medium | | selective medium | | |
| --- | --- | --- | --- | --- | --- |
| Strains | cell number (log cfu/ml) | colony diamater | NaCl amount | cell number (log cfu/ml) | Colony diamater |
| *A. acidoterrestris* DSM 3922 | 5.81±0.09 | 5 mm | 0.5% | 5.78±0.02 | 3 mm |
| *A. acidoterrestris* CB-1 | 6.51±0.11 | 6 mm | 1% | 6.46±0.05 | 4 mm |
| *A. acidoterrestris* C8 | 6.32±0.14 | 6 mm | 1% | 6.28±0.06 | 4 mm |
| *A. acidoterrestris* C24 | 7.04±0.19 | 5 mm | 0.8% | 7.11±0.09 | 3 mm |
